# A Rare Compound Heterozygous NAGLU Gene Mutation in Two Siblings with Mucopolysaccharidosis type Iiib

**DOI:** 10.30699/ijp.2025.2055938.3426

**Published:** 2025-11-11

**Authors:** Laleh Vahedi-Larijani, Maryam Sotoudeh Anvari, Alireza Biglari, Maryam Nabati, Hosna Banihashemi, Marzie Mohammadi Kharkeshi

**Affiliations:** 1 *Department of Pathology, Faculty of Medicine, Mazandaran University of Medical Sciences, Sari, Iran*; 2 *Department of Molecular Pathology, Children Medical Center, Tehran University of Medical Sciences, Tehran, Iran*; 3 *Faculty of Medicine, Tehran University of Medical Sciences, Tehran, Iran*; 4 *Department of Cardiology, Faculty of Medicine, Mazandaran University of Medical Sciences, Cardiovascular Research Center, Sari, Iran*; 5 *Faculty of Medicine, Babol University of Medical Sciences, Babol, Iran*; 6 *Mazandaran University of Medical Sciences, Pathology Research Center, Sari, Iran*

**Keywords:** Genetic Diseases, Mucopolysaccharidosis Type IIIB, Inborn Errors of Metabolism, Glycosaminoglycan, Intellectual Disabilities

## Abstract

**Background & Objective:**

Mucopolysaccharidosis (MPS) type III, or Sanfilippo syndrome, is an autosomal recessive lysosomal storage disorder caused by mutations in genes encoding enzymes responsible for glycosaminoglycan (GAG) degradation. This case report describes two siblings with MPS type IIIB who exhibit a rare compound heterozygous mutation in the *NAGLU* gene.

**Case Presentation:**

A 7-year-old girl and her 4-year-old brother were referred for evaluation due to learning disabilities, aggressiveness, and coarse facial features. Enzyme assay using tandem mass spectrometry on dried blood spots in both siblings revealed absent N-acetyl-α-glucosaminidase activity.

**Conclusion:**

Targeted sequencing confirmed the diagnosis, identifying two heterozygous mutations—an in-frame insertion and a missense mutation—in exon 3 of the *NAGLU* gene: c.214_237dup (p.Ala72_Gly79dup) and c.625A>C (p.Thr209Pro). This rare genetic finding in two siblings with Sanfilippo syndrome type B underscores the importance of precise mutation identification. Accurate characterization of defective gene variants may provide insights into potential targets for gene therapy in monogenic disorders.

## Introduction

Mucopolysaccharidosis type III (MPS III), also known as Sanfilippo disease, comprises four autosomal recessive disorders (types A–D) identified by mutations in genes encoding enzymes involved in the degradation of heparan sulfate glycosaminoglycan (1). The lifetime risk at birth for all MPS III subtypes ranges from 0.17 to 2.35 per 100,000 live births (2). The disorder exhibits considerable molecular heterogeneity, and 156 mutations have been described in the *NAGLU* gene to date (2,3). Homozygous or compound heterozygous mutations in the *N*-alpha-acetylglucosaminidase (*NAGLU*) gene, located on chromosome 17q21, are responsible for Sanfilippo syndrome type B (MPS IIIB) (OMIM #252920) (4).

Accumulation of mucopolysaccharides causes lysosomal swelling, disrupts the autophagosome–lysosome axis, and leads to severe central nervous system degeneration. This process results in cognitive dysfunction characterized by periods of aggressiveness and hyperactivity, which ultimately progress to neurological deterioration, severe dementia, and loss of motor skills (5,6).

MPS IIIB (Sanfilippo syndrome type B) is a lysosomal storage disorder caused by a deficiency of α-*N*-acetylglucosaminidase (*NAGLU*), leading to the accumulation of heparan sulfate predominantly in the central nervous system. Affected children are typically asymptomatic at birth but begin to show developmental slowing between 1 and 4 years of age, initially presenting with mild developmental and speech delays. This is followed by severe behavioral disturbances such as hyperactivity, restlessness, temper tantrums, aggressive outbursts, and sleep disturbances, which occur in up to two-thirds of patients (7). Progressive intellectual decline culminates in profound dementia, movement disorders, and eventual loss of ambulation, with many patients surviving into the second or third decade, particularly in attenuated phenotypes. Somatic manifestations are generally mild and may include coarse facial features, macrocephaly, hepatomegaly, and mild dysostosis multiplex (8).

Molecular analyses have demonstrated remarkable allelic heterogeneity, with more than one hundred distinct *NAGLU* mutations—including predominantly missense variants as well as insertions, deletions, and nonsense mutations—distributed throughout the gene. Only a few recurrent alleles (e.g., p.R643C, p.R297X) occur with appreciable frequency in certain populations, and mutation hotspots may correlate with disease severity or attenuated phenotypes (9). Despite extensive mutation cataloging and limited genotype–phenotype observations, the relationship between specific *NAGLU* variants—particularly novel compound heterozygotes—and the wide spectrum of clinical severity remains poorly understood. This represents a critical knowledge gap for prognostic counseling and the stratification of emerging enzyme- and gene-based therapies (10).

In this case report, we present a rare compound heterozygous mutation in the *NAGLU* gene identified in two siblings with MPS IIIB.

## Case Presentation

Two siblings, a 7-year-old girl and her 4-year-old brother, were referred to our hospital for evaluation of hereditary metabolic disorders. They had an older brother, aged 13 years, who was clinically healthy. Neither the brother nor their unrelated parents exhibited any signs or symptoms suggestive of a metabolic disorder.

### Case 1: Clinical Features

The proband, a 7-year-old girl, was referred for evaluation of a suspected genetic metabolic disorder. She was born at term following an uncomplicated vaginal delivery, with an unremarkable neonatal and infancy course. At birth, a mild foot deformity was noted and attributed to oligohydramnios-related complications. Her mother also reported mild abdominal distension during infancy, presumed secondary to overfeeding, which resolved gradually. Early developmental milestones, including gross motor and language skills, were achieved appropriately until approximately 2.5 years of age.

Beginning around age 2.5 years, she exhibited progressive facial coarsening, characterized by thickened lips, tongue, eyebrows, and eyelashes. Other physical findings included mild hirsutism and an umbilical hernia. Concurrently, she developed speech disturbances, including stuttering and difficulty articulating /a/ and /s/ sounds. These were initially associated with nocturnal terrors following a psychologically stressful event (a hand burn sustained at daycare). The symptoms were initially attributed to psychological causes, and she was referred for speech and occupational therapy. Despite intervention, her condition progressively worsened.

**Fig. 1:**
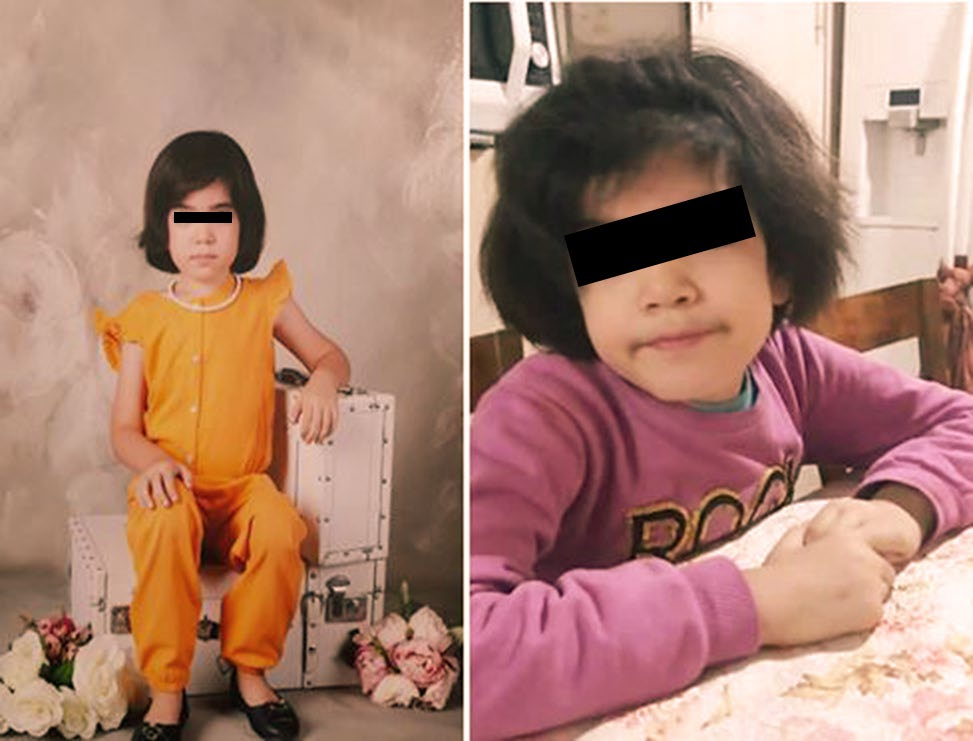
Photograph of female sibling shows mild coarseness of face and hirsutism

By age 5, she manifested worsening neurobehavioral symptoms resembling autism spectrum disorder, including aggressive behavior, attention-deficit/hyperactivity disorder, intellectual disability, and restlessness. She also developed urinary and fecal incontinence, and her speech regressed to incoherent, repetitive phrases. There was no history of seizures or loss of consciousness.

**Fig. 2:**
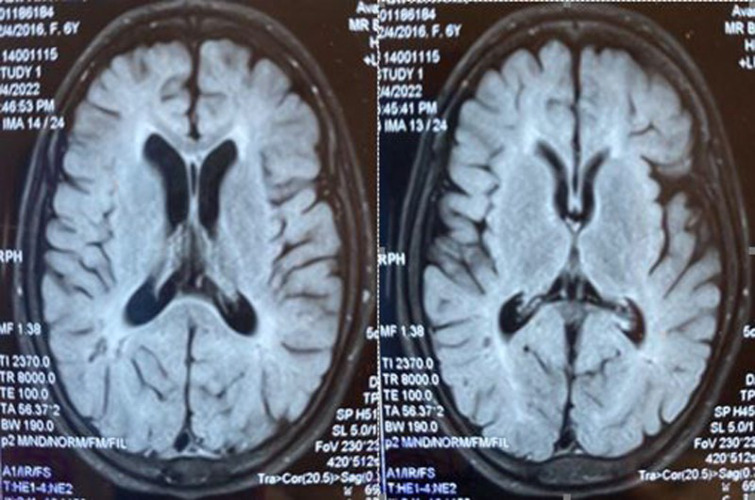
Female sibling axial T1 weighted brain MRI images show periventricular gliosis

Neurologic evaluation revealed borderline intellectual functioning. On physical examination, the patient had short stature, coarse facial features, mild macroglossia, a long philtrum, mild hirsutism, an umbilical hernia, and a persistent deformity of the left foot (Figure 1). Electroencephalography (EEG) demonstrated paroxysmal epileptiform discharges. Brain magnetic resonance imaging (MRI) revealed periventricular gliosis, cerebral volume loss with thinning of the corpus callosum, and prominent perivascular spaces (Figure 2). Pharmacologic management with risperidone and sodium valproate yielded minimal improvement in behavioral symptoms. Urinalysis was positive for mucopolysaccharides on metabolic screening.

### Case 2: Clinical Features

**Fig. 3:**
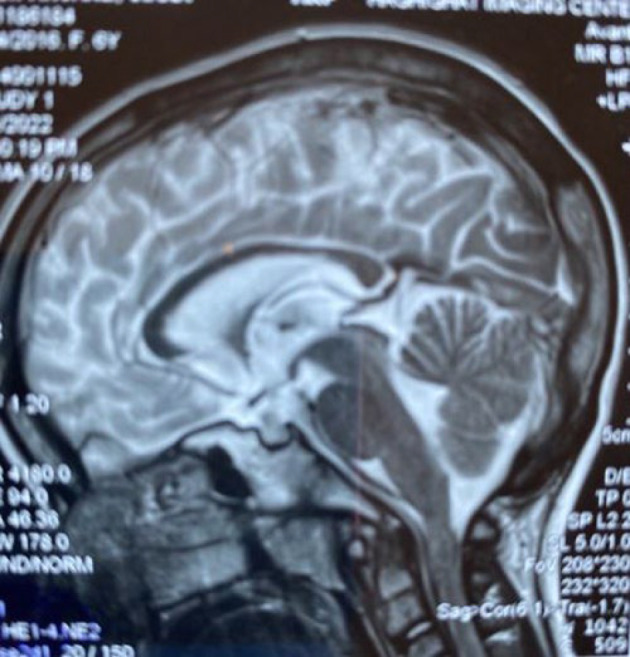
Female sibling sagittal T2 weighted brain MRI image shows thinning of corpus callosum

The younger sibling, a 4-year-old boy, was referred for metabolic and developmental evaluation. He exhibited global developmental delay, with cognitive and learning impairments more pronounced than those of his sister. Physical examination revealed coarse facial features and short stature consistent with a lysosomal storage disorder phenotype (Figure 3). Since diagnosis, he has been enrolled in specialized rehabilitation services at an autism clinic, which has helped moderately slow his neurodevelopmental decline.

**Fig. 4:**
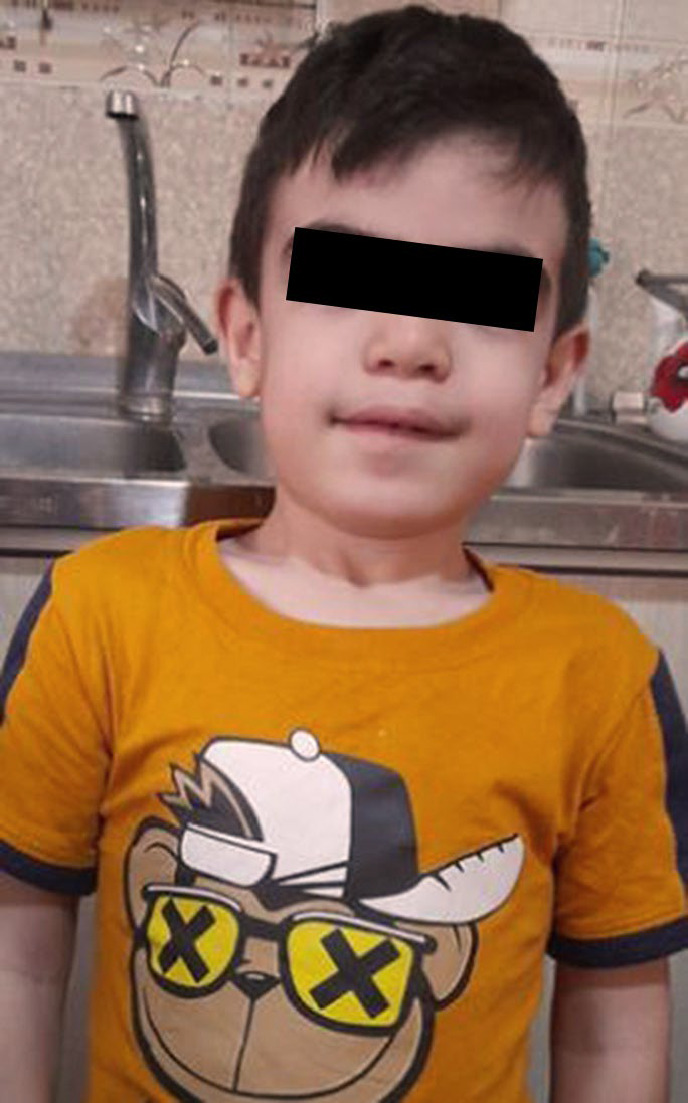
Male sibling axial T2 weighted brain MRI image shows periventricular

Neuroimaging by brain MRI demonstrated abnormal myelination, with persistent unmyelinated white matter regions in both temporal lobes and periventricular areas (Figure 4). Abdominal ultrasonography revealed hepatosplenomegaly, with spleen dimensions of 98 × 43 mm and a right hepatic lobe measuring 137 mm, both with homogeneous echogenicity.

### Metabolic and Genetic Studies

Both siblings underwent comprehensive metabolic and genetic testing. Biochemical evaluation showed a positive urinary glycosaminoglycan (GAG) screening by Berry spot test and slightly increased methylmalonyl/3-hydroxy-isovalerylcarnitine (C4DC/C5OH) levels on tandem mass spectrometry (MS/MS) of dried blood spots (DBS). Amino acid profiles by high-performance liquid chromatography (HPLC), thyroid function, complete blood count, and homocysteine levels were within normal limits.

Enzyme activity assay by tandem MS/MS on DBS and peripheral leukocytes demonstrated absent *N*-acetyl-α-glucosaminidase activity (0 µmol/L/h), while activities of enzymes related to MPS types I, II, IIIB, IVA, V, and VII were normal. The mother’s *N*-acetyl-α-glucosaminidase activity was at the lower limit of normal (0.5 µmol/L/h; cutoff >0.5 µmol/L/h). These results established the diagnosis of MPS IIIB in both siblings.

For molecular confirmation and carrier detection, 5 mL of peripheral blood was collected in ethylenediaminetetraacetic acid (EDTA)-containing tubes, and genomic DNA was extracted from leukocytes. Whole-exome sequencing (WES) was performed on an Illumina NovaSeq 6000 platform with 100× on-target coverage and a read length of 151 bp. WES initially identified a heterozygous variant of uncertain significance (VUS) in *NAGLU* (NM_000263.4: exon 3: c.625A>C; p.Thr209Pro). Although consistent with a potential *NAGLU*-related disorder, its heterozygous state did not fully explain the clinical phenotype, possibly due to WES coverage limitations.

Subsequent targeted gene sequencing for MPS-related genes with higher coverage confirmed two heterozygous variants in *NAGLU*: an in-frame insertion (c.214_237dup; p.Ala72_Gly79dup) and a missense variant (c.625A>C; p.Thr209Pro) (11). Detection of these two variants confirmed the diagnosis of MPS IIIB. Targeted sequencing in the mother revealed a heterozygous c.214_237dup (p.Ala72_Gly79dup) mutation, consistent with carrier status. 

**Fig. 5:**
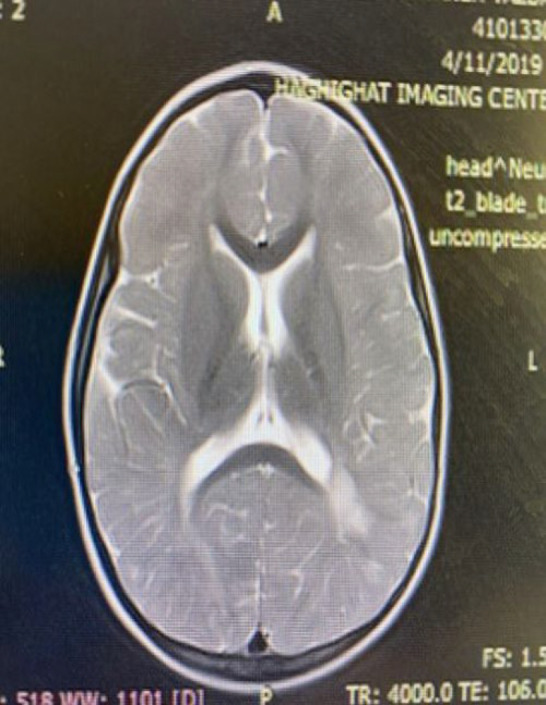
. Male sibling axial T2 weighted brain MRI image shows periventricular gliosis

### Diagnostic Workup

#### Biochemical Studies

Both siblings underwent extensive metabolic testing. Urinary glycosaminoglycan (GAG) screening via the Berry spot test was positive, supporting a diagnosis of mucopolysaccharidosis. Tandem mass spectrometry (MS/MS) on dried blood spots showed slightly elevated levels of methylmalonyl-/3-hydroxy-isovalerylcarnitine (C4DC/C5OH). Amino acid analysis by high-performance liquid chromatography (HPLC) demonstrated normal amino acid profiles. Additional laboratory investigations, including thyroid function tests, complete blood count, and homocysteine levels, were within normal limits.

### Enzyme Activity Assays

Enzyme activity was measured using tandem mass spectrometry (MS/MS) on dried blood spots and peripheral blood leukocytes. Both siblings exhibited undetectable N-acetyl-α-glucosaminidase activity (0 μmol/L/h). Enzyme activities for other mucopolysaccharidosis types—including MPS I, II, IIIA, IVA, V, and VII—were within normal reference ranges. The mother’s N-acetyl-α-glucosaminidase activity was at the lower limit of normal (0.5 μmol/L/h; cutoff > 0.5 μmol/L/h), consistent with carrier status.

### Genetic Analysis

Genomic DNA was extracted from peripheral blood leukocytes collected in ethylenediaminetetraacetic acid (EDTA) tubes. Initial whole-exome sequencing (WES), performed on an Illumina NovaSeq 6000 platform with 100× coverage and a 151-bp read length, identified a heterozygous variant of uncertain significance (VUS) in the NAGLU gene (NM_000263.4: exon 3, c.625A>C; p.Thr209Pro). Because of its heterozygous nature and inconclusive correlation with the clinical phenotype, further testing was pursued.

Subsequent targeted gene panel sequencing with higher depth and coverage, including all coding exons and flanking intronic regions of NAGLU, revealed two heterozygous mutations: an in-frame duplication c.214_237dup (p.Ala72_Gly79dup) and a missense variant c.625A>C (p.Thr209Pro). The detection of both mutations confirmed the diagnosis of MPS IIIB. Carrier testing in the mother demonstrated heterozygosity for the c.214_237dup (p.Ala72_Gly79dup) variant, supporting the autosomal recessive inheritance pattern.

### Treatment Plan and Prognosis

Following confirmation of the diagnosis, both patients were enrolled in a clinical trial involving 12 rounds of trehalose, a disaccharide with potential neuroprotective properties. As of the sixth injection, no measurable clinical improvement has been observed.

**Table 1:** summary table comparing key clinical features of both siblings

Clinical Feature	7-Year-Old Girl (Older Sibling)	4-Year-Old Boy (Younger Sibling)
Developmental Milestones	Achieved appropriate milestones up to 2.5 years; then gradual regression	Delayed milestones from early infancy; more severe learning disabilities than sister
Language Skills	Appropriate language development until 2.5 years; progressive speech deterioration afterward	Severe learning and comprehension disorders; speech impairment implied through developmental delay
Behavioral Features	Autism-like behaviors, aggression, ADHD, restlessness by age 5	Diagnosed with autism spectrum disorder; receiving rehabilitation
Neurological Symptoms	Borderline IQ; no reported seizures; EEG: paroxysmal epileptiform discharges	brain MRI shows abnormal myelination
Physical Features	Coarse facial features, short stature, mild macroglossia, thickened lips/tongue/eyebrows/eyelashes, mild hirsutism, long philtrum, umbilical hernia, foot deformity	Coarse facial features, short stature, mild hirsutism, mild macroglossia, thickened lips/tongue/eyebrows/eyelashes
Urinary and Fecal Incontinence	Present	-
Neuroimaging (Brain MRI)	Periventricular gliosis, volume loss, corpus callosum thinning, prominent perivascular spaces	Unmyelinated white matter in bilateral temporal lobes and periventricular region, Periventricular gliosis
Abdominal Imaging (Ultrasound)		Hepatosplenomegaly (spleen 98×43 mm; liver right lobe 137 mm), both with homogeneous echo pattern
Therapies Received	Risperidone and sodium valproate (limited efficacy); speech & occupational therapy	Specialized rehabilitation services in autism clinic (moderate slower progression)
Urinary Metabolic Screening	Positive for mucopolysaccharidosis markers	-

## Discussion

The present case report describes two siblings diagnosed with Mucopolysaccharidosis type IIIB (MPS IIIB) who exhibited early neurodevelopmental concerns, including learning disabilities, aggressive behavior, and subtle somatic features such as coarse facial characteristics. Enzyme assay via tandem mass spectrometry confirmed a complete deficiency of α-N-acetylglucosaminidase activity in both siblings, while genetic analysis identified two novel heterozygous mutations in the NAGLU gene—a missense mutation (p.Thr209Pro) and an in-frame insertion (p.Ala72_Gly79dup)—establishing a diagnosis of compound heterozygous MPS IIIB.

To our knowledge, this is a rare report of such compound heterozygous mutations coexisting within the same family. While the in-frame duplication is likely pathogenic, the missense mutation remains classified as a variant of uncertain significance (VUS), underscoring the need for further functional validation. These findings expand the mutational spectrum of MPS IIIB and contribute to understanding the genetic basis of this rare disorder.

MPS III, also known as Sanfilippo syndrome, is a rare hereditary disorder that is characterized by severe central nervous system degeneration and somatic features. The α-N-acetylglucosaminidase enzyme is encoded by NAGLU gene and its deficiency is responsible for the appearance of MPS IIIB. The NAGLU gene is inserted on chromosome 17q21.2, spans 8.5 kb, and includes 6 exons. 156 mutations have been reported that 70.3% of total number of reported mutations were missense mutations. These different mutations cause a broad phenotypic spectrum in this form of syndrome (9, 12).

 The clinical and genetic features observed in the two siblings with Mucopolysaccharidosis type IIIB (MPS IIIB) in this report closely align with previously documented cases but also present unique aspects that contribute to the expanding phenotypic and genotypic spectrum of the disease. Clinically, the siblings exhibited early neurodevelopmental issues such as learning disabilities and behavioral disturbances, including aggressiveness, consistent with typical MPS IIIB presentations noted in the literature, where cognitive decline and behavioral problems manifest in early childhood. Their mild somatic features, including coarse facial characteristics, are also in agreement with prior reports of generally subtle somatic involvement in MPS IIIB. Genetically, these siblings harbor two novel heterozygous mutations in the NAGLU gene: an in-frame insertion (c.214_237dup: p.Ala72_Gly79dup) and a missense mutation (c.625A>C: p.Thr209Pro). While a broad allelic heterogeneity with numerous missense, nonsense, insertion, and deletion mutations has been well-established in MPS IIIB, these specific variants have not been extensively reported before, thereby enriching the mutation database. Previously reported compound heterozygous cases similarly underscore the challenge of correlating genotype with phenotypic severity, as mutation combinations result in variable clinical manifestations (13). 

MPS IIIB manifests a heterogeneous clinical course, with variable severity even within the same family, although most patients usually develop a milder form of the classical disorder (14). 

Trehalose, a disaccharide composed of two glucose molecules connected by an α, α-1,1-glucosidic bond, has garnered significant attention over the past few decades for its potential neuroprotective properties, particularly in animal models of neurodegenerative diseases such as Parkinson’s and Huntington’s diseases. Despite ongoing research, the precise mechanisms underlying trehalose’s neuroprotective effects remain unclear. A prominent theory suggests that trehalose exerts its protective effects by inducing autophagy, which facilitates the clearance of protein aggregates. This hypothesis is supported by several animal studies demonstrating increased autophagy and reduced protein aggregates following trehalose administration in models of neurodegenerative diseases. The rationale for using trehalose in mucopolysaccharidosis lies in its potential neuroprotective effect by inducing autophagy, which may help clear accumulated cellular waste products and protein aggregates that contribute to neurodegeneration in the disease (15).

Gene therapy which transfers the wild-type gene to correct the genetic defect may be the best therapeutic option. The insertion of the normal gene into hundreds of millions of cells that contain the defective gene is performed by non-pathologic adenovirus-based vectors (16). On the other hand, enzyme replacement therapy is currently the most successful treatment for the non-neurological symptoms of lysosomal storage diseases (17). Although these treatment modalities are still in experimental stages and are only being tested in animal models, they seem promising approaches to achieve long-lasting treatment for MPS III patients (12). 

The underscores limitations of WES in detecting certain types of mutations, such as duplications/ insertions or deep intronic variants, highlights the need for complementary sequencing approaches in genetic diagnosis.

For future research, it is imperative to conduct functional studies to evaluate the pathogenicity of identified variants, especially those of uncertain significance. Expanded genetic screening, including whole-genome sequencing, could identify additional regulatory or non-coding mutations and clarify genotype–phenotype relationships. Longitudinal natural history studies with larger cohorts would improve understanding of disease progression and variability. Investigation of potential modifier genes or epigenetic factors may elucidate mechanisms underlying clinical heterogeneity. Finally, rigorous clinical evaluation of novel therapies including trehalose, gene therapy, and enzyme replacement should continue, with emphasis on neurological outcome measures.

## Conclusion

In conclusion, this case report describes two siblings with Mucopolysaccharidosis type IIIB harboring novel compound heterozygous mutations in the NAGLU gene, expanding the mutational spectrum associated with the disease. Their clinical presentation of early neurodevelopmental delay and behavioral disturbances aligns with typical MPS IIIB features, while the lack of response to trehalose therapy underscores the ongoing challenges in treating this progressive neurodegenerative disorder. These findings highlight the importance of comprehensive genetic analysis for accurate diagnosis and contribute valuable data toward understanding genotype-phenotype correlations and informing future therapeutic strategies.

## Abbreviations

MPS: Mucopolysaccharidosis

GAG: Glycosaminoglycan

NAGLU: N-alpha acetylglucosaminidase

MRI: Brain magnetic resonance imaging

DBS: Dried blood spots

HPLC: High-performance liquid chromatography

EDTA: Ethylenediamine tetra-acetic acid

WES: Whole exome sequencing

VUS: Variant of Uncertain Significance 

## References

[1] Anikiej-Wiczenbach P, Manski A, Milska-Musa K, Limanowka M, Wierzba J, Jamsheer A (2022). Highly diverse phenotypes of mucopolysaccharidosis type IIIB sibling patients: effects of an additional mutation in the AUTS2 gene. J Appl Genet.

[2] Zelei T, Csetneki K, Voko Z, Siffel C (2018). Epidemiology of Sanfilippo syndrome: results of a systematic literature review. Orphanet J Rare Dis.

[3] Schmidtchen A, Greenberg D, Zhao HG, Li HH, Huang Y, Tieu P (1998). NAGLU mutations underlying Sanfilippo syndrome type B. Am J Hum Genet.

[4] Hettiarachchi D, Nethikumara N, Pathirana B, Weththasigha K, Dissanayake WDN, Dissanayake VHW (2018). A novel mutation in the NAGLU gene associated with Sanfilippo syndrome type B (mucopolysaccharidosis III B). Clin Case Rep.

[5] Champion KJ, Basehore MJ, Wood T, Destree A, Vannuffel P, Maystadt I (2010). Identification and characterization of a novel homozygous deletion in the alpha-N-acetylglucosaminidase gene in a patient with Sanfilippo type B syndrome (mucopolysaccharidosis IIIB). Mol Genet Metab.

[6] Veraldi N, Quadri ID, de Agostini A (2022). Characterization of a spontaneous cell line from primary mouse fibroblasts as a model to study Sanfilippo syndrome. The International Journal of Biochemistry & Cell Biology..

[7] Albar RF, AlQurashi RA, Naaman N, Alghamdi A, Alghamdi SK, Aljohani K (2022). A Novel Mutation in the NAGLU (N-Acetyl-Alpha-Glucosaminidase) Gene Associated With Mucopolysaccharidosis Type III-B in a Saudi Girl. Cureus.

[8] Ozkinay F, Emecen DA, Kose M, Isik E, Bozaci AE, Canda E (2021). Clinical and genetic features of 13 patients with mucopolysaccarhidosis type IIIB: Description of two novel NAGLU gene mutations. Mol Genet Metab Rep..

[9] Beesley CE, Jackson M, Young EP, Vellodi A, Winchester BG (2005). Molecular defects in Sanfilippo syndrome type B (mucopolysaccharidosis IIIB). J Inherit Metab Dis.

[10] Weber B, Guo XH, Kleijer WJ, van de Kamp JJ, Poorthuis BJ, Hopwood JJ (1999). Sanfilippo type B syndrome (mucopolysaccharidosis III B): allelic heterogeneity corresponds to the wide spectrum of clinical phenotypes. Eur J Hum Genet.

[11] Alaei MR, Kheirkhahan M, Talebi S, Davoudi-Dehaghani E, Keramatipour M (2019). Once in a blue moon, a very rare coexistence of glutaric acidemia type I and mucopolysaccharidosis type IIIB in a patient. Iranian Biomedical Journal.

[12] Andrade F, Aldámiz‐Echevarría L, Llarena M, Couce ML (2015). Sanfilippo syndrome: Overall review. Pediatrics International.

[13] Cross EM, Hare DJ (2013). Behavioural phenotypes of the mucopolysaccharide disorders: a systematic literature review of cognitive, motor, social, linguistic and behavioural presentation in the MPS disorders. J Inherit Metab Dis.

[14] Valstar MJ, Bruggenwirth HT, Olmer R, Wevers RA, Verheijen FW, Poorthuis BJ (2010). Mucopolysaccharidosis type IIIB may predominantly present with an attenuated clinical phenotype. J Inherit Metab Dis.

[15] Lee HJ, Yoon YS, Lee SJ (2018). Mechanism of neuroprotection by trehalose: controversy surrounding autophagy induction. Cell Death Dis.

[16] Di Domenico C, Villani GR, Di Napoli D, Nusco E, Cali G, Nitsch L (2009). Intracranial gene delivery of LV-NAGLU vector corrects neuropathology in murine MPS IIIB. Am J Med Genet A.

[17] Brady RO (2006). Enzyme replacement for lysosomal diseases. Annu Rev Med.

